# Associations Between Neighborhood Environment, Childhood Adversity, and Cancer Risk: A Geospatial Analysis

**DOI:** 10.1002/cam4.71331

**Published:** 2025-10-29

**Authors:** Tesla D. DuBois, Shannon M. Lynch, Jayme Banks, David B. Sarwer, Krista Schroeder

**Affiliations:** ^1^ Sidney Kimmel Comprehensive Cancer Center – Jefferson Health Philadelphia Pennsylvania USA; ^2^ Department of Geography Temple University Philadelphia Pennsylvania USA; ^3^ Department of Cancer Prevention and Control Fox Chase Cancer Center Philadelphia Pennsylvania USA; ^4^ Lewis Katz School of Medicine Temple University Philadelphia Pennsylvania USA; ^5^ Department of Prevention, Intervention, & Trauma School District of Philadelphia Philadelphia Pennsylvania USA; ^6^ Barnett College of Public Health Temple University Philadelphia Pennsylvania USA

**Keywords:** built environment, cancer, crime, spatial analysis, trauma

## Abstract

**Objective:**

Using a socio‐ecological perspective that includes consideration for the effects of the built environment, this study investigated the association between neighborhood‐level measures of childhood trauma, adult cancer prevention behaviors, and cancer mortality in Philadelphia.

**Methods:**

Cancer registry data and five neighborhood‐level risk factors, including three measures related to trauma and two indices related to adherence to cancer prevention guidelines, were utilized. Multivariate linear regression analyses were performed to assess how risk factors relate to variation in cancer mortality. Associations between each of the neighborhood risk factors and high cancer mortality were identified and visualized geospatially.

**Results:**

Trauma and adherence to cancer prevention guidelines together accounted for 50% of the variation in neighborhood cancer mortality in Philadelphia. Neighborhoods near each other were likely to have common prevalent risk factors, and the majority of neighborhoods with the highest cancer mortality rates were also high in trauma measures.

**Conclusions:**

Results expand upon prior research in this area to the neighborhood level, suggesting that neighborhoods with high cancer mortality are more likely to have high levels of trauma and low cancer prevention behaviors. Neighborhood‐level measures of trauma can be used to prioritize and tailor trauma‐informed cancer prevention efforts.

## 1 Introduction

Childhood trauma, as measured by Adverse Childhood Experiences (ACEs), is linked to poor physical health, including increased cancer risk, in adulthood. For example, an increased ACE score (e.g., more ACEs) is associated with chronic morbidities including cardiovascular disease, diabetes, and respiratory disorders, as well as obesity and chronic pain [1, 2, 3, 4]. Modifiable factors may attenuate the relationship between ACEs and cancer in adulthood. For example, physically active individuals may experience weaker associations between ACEs and poor physical health outcomes [2].

The association between childhood trauma and adult cancer is not well understood. At the individual level, two possible pathways have been suggested [5, 6]. First, childhood trauma has enduring effects on an individual's ability to engage in health promotion activities throughout their lifespan, such that individuals who have experienced traumatic events during their childhood are more prone to engaging in behaviors that pose health risks, such as smoking, alcohol and drug use, less healthy diet, and physical inactivity [3]. The cumulative impact of multiple traumatic events further heightens the probability of engaging in such behaviors [4, 7, 8]. Modifiable factors, including adherence to cancer prevention guidelines and health promotion activities, may also explain some of the association between childhood trauma and cancer risk [9].

The second possible pathway between childhood trauma and adult cancer is related to the biological impact of chronic stress [10]. ACEs are associated with an increased allostatic load, a measure of the cumulative biological burden of persistently high‐stress exposure [11]. A high allostatic load has been linked to increased cancer risk via dysregulation of the hypothalamic–pituitary–adrenal axis and the sympathetic adrenal medullary pathways that lead to physiologic damage, changes in gene regulation, and increases in pro‐inflammatory cytokines such as Tumor Necrosis Factor‐α [12, 13]. For example, in a recent study of over 3000 multiethnic women, the highest allostatic load was associated with a 64% increased risk of overall cancer incidence, even after adjusting for demographics, health behaviors, and socioeconomic factors [14]. A nationally representative study of over 17,000 US residents found a 23% increased risk of cancer death for adults with high allostatic load and an increased risk for adults with obesity [15].

Trauma is complex and can be related to a diverse source of exposures. More specifically, childhood trauma can result from a onetime event (e.g., sexual assault), ongoing events (e.g., living with a parent who has substance use disorder), or chronic exposures (e.g., experiencing identity‐based discrimination across childhood). A socio‐ecological framework, which takes into account multiple levels of influence and the complex interactions between individuals, communities, and their environment, can be used to investigate the associations between childhood trauma and cancer outcomes later in life [16]. Importantly, ACEs can occur across levels of ecology, including the individual, household, neighborhood, school, or societal level [17, 18, 19]. Therefore, various measures of trauma may be associated with cancer burden at the area level.

Factors in the neighborhood environment are associated with trauma exposure [18]. For example, recent work highlights the potential for peer‐ and neighborhood‐level exposures such as experiencing bullying or living in an unsafe neighborhood to be traumatizing for youth [20]. Additionally, neighborhood factors such as lack of access to healthcare or substance use disorder treatment might make it more challenging to engage in health habits that can buffer elevated cancer risk associated with trauma exposure. Thus, an examination of collective neighborhood factors associated with trauma is needed to understand whether and how this level of ecology can influence ACEs–cancer associations.

A neighborhood‐level variable that has received limited attention in this area is the school setting. In Philadelphia, the majority of school‐aged children attend their local “neighborhood” or “catchment” school, which is a school that serves the students residing within the geographic bounds of a designated area. Such schools are intrinsically impacted by and impact their local community. Further, they provide resources that enhance student resilience and play a crucial role in fostering a sense of belonging [21, 22]. Therefore, a comprehensive investigation of trauma may include school‐level factors associated with children who have experienced trauma. For example, students who have experienced trauma often are challenged to attend school regularly; they also are likely to move between schools more than those who have not [23, 24]. Additionally, behaviors associated with trauma frequently result in disciplinary action [23, 24].

Another variable of importance, particularly in Philadelphia, is gun violence. In 2017, 1256 citizens of Philadelphia were impacted by gun violence [25]. Even for those who are not directly impacted, living among high rates of violent crime can generate chronic stress and trauma within a community [26, 27, 28, 29]. Therefore, in a socio‐ecological model, violent crime may serve as an important indicator of potential trauma at a neighborhood level that could relate to cancer burden.

In Philadelphia, significant geographic disparities in cancer burden exist across various neighborhoods [30]. Specifically, there are differences by neighborhood in cancer mortality and in behaviors that can affect cancer risk, including smoking, diet, and physical activity. Understanding the relationship between psychological trauma and cancer burden (defined in terms of mortality and health behaviors) at the neighborhood level is imperative to address cancer and cancer disparities. Thus, the aim of this study was to explore the association between childhood trauma and adult cancer mortality within the city of Philadelphia and identify neighborhood profiles that incorporate both trauma and adherence to cancer prevention guidelines, with the goal of informing future research and prevention efforts.

## 2 Methods

### 2.1 Study Design

This was a cross‐sectional, secondary analysis of publicly available spatial and surveillance data. The aim was to examine associations between cancer mortality and selected neighborhood‐level social, behavioral, and environmental risk factors.

### 2.2 Study Area

The setting for this study was Philadelphia, Pennsylvania. The city is a densely populated and socioeconomically and demographically diverse urban area with 1.6 million residents, segmented into 46 neighborhoods for this analysis. Each neighborhood was comprised of an agglomeration of 4–16 bordering census tracts (CTs), collectively housing approximately 31,000 residents [30]. Moreover, these neighborhood boundaries were intentionally designed to reflect communities that are consistent with commonly recognized Philadelphia neighborhoods, setting them apart from geographic units developed for administrative purposes like CTs or zip codes.

### 2.3 Measures

The outcome of interest was cancer mortality. The five exposure measures of interest crossed the lifespan, capturing trauma measures in childhood and adult cancer prevention and health promotion. Given the inclusion of all‐cause cancer mortality, which comprises both rapidly and slowly progressing cancers, a consistent exposure window could not be defined. As such, available contemporary data were used to characterize neighborhood conditions and explore patterns of co‐occurrence. Each is detailed below. The exposure measures were converted to a standard score (*z*‐score) [31] in order to assess the relative position of a neighborhood compared to the other neighborhoods on the given measure.

#### 2.3.1 Outcome: Neighborhood‐Level Cancer Mortality

Cancer mortality was operationalized as the total number of cancer deaths in Philadelphia per 100,000 residents, per all cancer‐related deaths recorded in the PA State Cancer Registry, 2012–2016. Residential addresses of these individuals were geocoded to the CT using ESRI Business Analyst 2016 Geocoder. Age‐specific mortality rates were calculated using 5‐year CT population estimates from the US Census, American Community Survey (ACS) [32]. Population and cancer mortality counts at the CT level were aggregated to the neighborhood level before generating neighborhood mortality rates [30].

#### 2.3.2 Exposure Metrics Related to Trauma

##### 2.3.2.1 Neighborhood‐Level Childhood Trauma

Neighborhood‐level childhood trauma was operationalized via a neighborhood‐level Adverse Childhood Experiences (ACE) Index [18]. A Neighborhood ACEs Index (NAI) can be understood as a single‐item area‐level measure that captures neighborhood factors associated with individual‐level trauma exposure. The NAI was created by using Bayesian index regression to capture the association between 25 neighborhood factors and individual‐level trauma exposure. Neighborhood factors included in the index include diverse neighborhood characteristics potentially associated with trauma exposure, such as violent crime, economic deprivation, racial segregation, high rates of poor mental health, alcohol outlet access, lack of access to substance use treatment, and more. The resulting NAI is a continuous variable; a neighborhood with a higher NAI score has more factors associated with trauma. The NAI was previously created and validated for each Philadelphia CT [18]. For the present analysis, Neighborhood ACEs Index scores were agglomerated from CT to neighborhood. CT weights were calculated based on each CT's contribution to each neighborhood's total population. Subsequently, these weights adjusted CT‐level neighborhood trauma scores, weighted scores were summed within each neighborhood, and a standard score was calculated from the result. This process yielded a neighborhood‐level measure of childhood trauma based on factors related to the neighborhood environment, hereafter referred to as “NAI.”

##### 2.3.2.2 Neighborhood‐Level School Trauma

A novel school trauma index was created specifically for this analysis using publicly available data from the School District of Philadelphia [33]. School‐level discipline, attendance, and retention measures were selected for inclusion in the index based on their known association with trauma [23, 34]. Out‐of‐school suspension (% of Students Receiving Zero Out‐of‐School Suspensions) and student attendance (% of Students Attending Less Than 80% of Instructional Days) were based on data from the 2021–2022 school year, while a measure of student retention (a school district measure of how well the school did on student retention) was from the 2018–2019 school year.

To attribute school‐level measures to neighborhoods, a three‐step process was required. First, the area of overlap between the 161 school catchment areas (the geographic bounds designating the resident population each school serves) and the 46 neighborhoods was calculated. Second, for each neighborhood, the proportion of area covered by each catchment was used as a weight in attributing school measures to the neighborhood level. Third, weighted contributions were summed within the neighborhood.

The three measures of attendance, retention, and suspension were combined into a single index following the methods used for the exposure metrics related to adult cancer described below [35]. For each indicator, standard scores were calculated, and then neighborhoods that had higher or lower rates compared to the city overall were identified using the first and fourth quartiles. Values of −1 were assigned to those less favorable than the city overall, 1 for those more favorable, and 0 for neighborhoods that were not at either extreme. Within each neighborhood, the values of the three measures were summed, and *z*‐scores were calculated across the set of observations, resulting in a neighborhood‐level school‐based trauma indicator.

##### 2.3.2.3 Neighborhood‐Level Exposure to Violent Crime

The rate of violent crimes, including murder/homicide, aggravated assault, robbery, and rape per 100,000 people was also included. This was obtained from the Philadelphia Police Department for the year 2017. To generate the neighborhood‐level crime rate, crime events occurring within the neighborhood's borders were summed and normalized by the total number of residents contributed by the CTs encompassed within each neighborhood, using 2017 US Census population data. A standard score was calculated to be able to compare each neighborhood relative to the mean [30]. While violent crime is one of the 25 measures in the NAI, it was examined separately to assess whether a single, widely available measure and a measure that we hypothesized to be particularly salient would yield similar insights.

#### 2.3.3 Exposure Metrics Related to Adult Cancer

##### 2.3.3.1 Neighborhood Adult Cancer Prevention Behaviors—Guideline Adherence

Cancer prevention behaviors are measured in terms of guideline adherence. “Guideline adherence,” was operationalized as a neighborhood or area‐level measure of adherence to cancer prevention guidelines by the American Cancer Society as developed by members of the study team and collaborators (DuBois et al. [35]). Drawing from multiple data sources (the Philadelphia Health Management Corporation, the American Census, the Philadelphia Police Department, the City of Philadelphia, the Trust for Public Land, and the United States Department of Agriculture) between the years 2015 and 2018, the index includes seven measures related to five recommendations for individuals regarding healthy behaviors. These include maintaining a healthy weight, being physically active, eating a healthy diet, limiting alcohol intake, and avoiding tobacco [36]. There also are six measures related to two recommendations for communities—including accessibility to healthy foods and providing a safe environment for physical activity. The final measure is a standard score that incorporates the 13 individual measures that are included in the seven recommendations.

##### 2.3.3.2 Neighborhood Adult Health Promotion Score

The cancer prevention measure related to health promotion, hereafter referred to as “health promotion,” was operationalized as a neighborhood‐level measure of cancer screening and community health. Drawing on data provided by the Philadelphia Health Management Corporation and the City of Philadelphia between the years 2016–2018, the index includes a self‐report measure of good health, a measure of the availability of physicians, and a measure of rates of breast, colorectal, and cervical cancer screening. The final measure is a standard score that incorporates the five individual measures included in the three recommendations [35].

## 3 Analysis

Standard descriptive and exploratory statistics, such as measures of central tendency and variation, testing for normality, and visual examination of histograms, were executed. Subsequently, four steps were employed to assess whether and how psychological trauma and adult cancer behaviors and health promotion are associated with cancer mortality at the neighborhood level in Philadelphia. First, to determine whether neighborhoods near each other were statistically more likely to have similar values, tests of spatial autocorrelation were conducted. Second, to determine the extent to which measures are related to each other, a test of Pearson correlation between measures was employed. Third, to establish whether combinations of measures can be used to explain variation in cancer mortality, multivariate regression models were used. Fourth, to identify where in the city different combinations of risk factors might be driving high cancer mortality, a map was generated to illustrate how included measures overlap with high cancer mortality throughout the city. Each step is described in more detail below. All steps of this analysis were conducted using R Statistical Software (v4.3.1; R Core Team [37] ).

### 3.1 Step 1: Evaluating Spatial Dependencies

Neighborhood maps were generated to visualize the spatial distribution of each measure. These maps allowed for the identification of spatial patterns and trends, potentially revealing geographic variations in the data and informing hypothesis generation. To quantitatively assess the spatial relationships visualized in the maps, a Global Moran's *I* (Moran's *I*) statistic was employed, which produces results ranging from −1 to 1. A Moran's *I* value of 1 signifies clustering, indicating that neighborhoods near one another have more similar values. Conversely, a value of −1 implies dispersion, where similar observations tend to be dispersed throughout space. A Moran's *I* statistic of 0 indicates a random spatial distribution. Additionally, a test of significance for the Moran's *I* was conducted, with a *p*‐value threshold set at < 0.05 to identify statistically significant spatial autocorrelation. The presence of significant clustering (positive spatial autocorrelation) or dispersion (negative spatial autocorrelation) points to nonrandom spatial patterns in the data. Clustering often suggests a relationship between location and the given measure. In the context of measures related to humans, clustering is often influenced by local factors such as socioeconomic or environmental conditions [38].

### 3.2 Step 2: Testing for Correlations

A Pearson correlation coefficient was calculated for each of the six measures to quantitatively assess the strength and direction of the linear relationships between the variables. Only statistically significant correlations are reported below, with significance determined by a criterion of an associated *p*‐value of < 0.05.

### 3.3 Step 3: Explaining Variation in Cancer Mortality

In the third step of the analysis, a series of multivariate linear regression models was applied. Standard model diagnostics were assessed. Given the spatial nature of our research question, careful attention was paid to heteroscedasticity because if spatial autocorrelation was detected among model residuals, a spatial regression model (rather than ordinary least squares) would be considered as an alternative.

### 3.4 Step 4. Identifying Patterns of Overlap

The final phase of the analysis aimed to discern the unique combinations of risk factors prevalent in neighborhoods with the highest cancer mortality. First, a subset of neighborhoods, which included only those in the top quartile of cancer mortality, was identified. Second, membership in the least favorable quartile of each significant predictor (identified in Step 3) was determined for each high‐mortality neighborhood. (For example, does a given high‐mortality neighborhood also have low adherence to cancer prevention guidelines? Does it have high trauma compared to the city overall?) Next, high cancer mortality neighborhoods were categorized by which risk factors were prevalent. Categorization could refer to one or more risk factors. Finally, a map of high‐mortality neighborhoods representing each by its prevalent risk factor(s) was produced. This step aimed to visualize spatial patterns of risk within the city and shed light on how different neighborhoods may experience differentiated indicators associated with high cancer mortality.

## 4 Results

The neighborhood cancer mortality rates in Philadelphia ranged from 112.5 to 279 per 100,000 residents (mean 196 ± 33 deaths per 100,000 residents). Given their transformation into *z*‐scores, the standardized neighborhood risk factor indicators each had a mean close to zero and adhered to a normal distribution.

### 4.1 Step 1: Evaluating Spatial Dependencies

Moran's *I* test demonstrated significant spatial dependency for each measure, such that neighborhoods near each other are likely to have similar values. Maps (Figure S1) of each measure visualize its spatial distribution by displaying the 1st and 4th quartiles in dark purple and dark green. The neighborhoods shown in dark purple can be understood to have less favorable values of the given measure than the middle‐50th percentile neighborhoods (shown in white), whereas the neighborhoods shown in green have more favorable values. Visual inspection reveals overlap between measures with regard to the areas of the city that have neighborhoods at either extreme.

### 4.2 Step 2: Testing for Correlations

**FIGURE 1:**
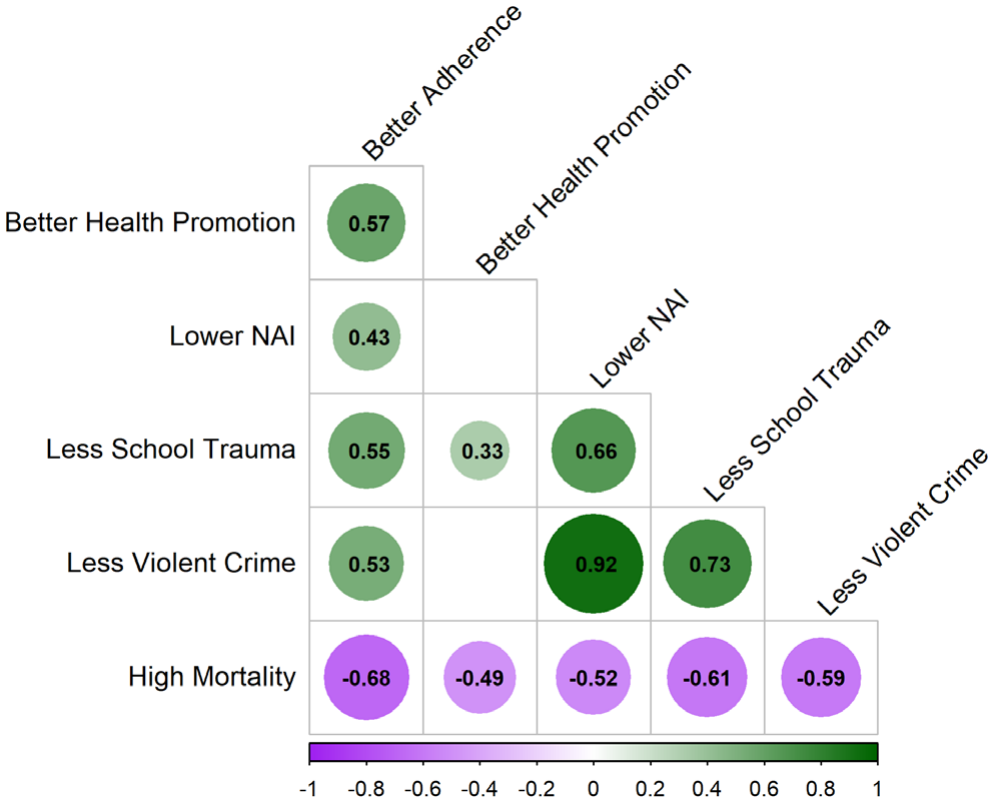
Correlation plot of exposure and outcome measures. This correlation plot shows significant correlations only between high mortality and favorable neighborhood characteristics (better health promotion, lower NAI, less school trauma, and less violent crime). Coefficients indicating positive correlations are indicated with green circles and those indicating inverse associations (negative correlations) are indicated with purple circles. The strength of the correlation is indicated numerically (a number further from 0) and with the shade and size of the colored circle whereby a darker shade and larger size indicates a stronger correlation.

As suggested by the overlap observed in the maps of indicators (Figure S1), many of the risk factors were correlated with each other and with cancer mortality (Figure 1). The three trauma indicators (Child, School, and Crime) were all positively correlated with each other. Violent crime was highly correlated with NAI (0.92), which was unsurprising as the NAI includes a measure of violent crime and that crime exposure is associated with risk for and experience of trauma. More interesting, however, was that the correlation was stronger between school trauma and violent crime (0.73) than between school trauma and NAI (0.66). Health promotion was not significantly correlated with violent crime or NAI, although it was correlated with school trauma (0.33), such that neighborhoods with better health promotion also tend to have fewer indicators of trauma at the school level. Health promotion was positively associated with guideline adherence (0.53). Better adherence was correlated with less school trauma (0.55), lower NAI (0.43), and less crime (0.53).

There was a significant inverse association between all favorable indicators (better health promotion, better guideline adherence, less school trauma, less violent crime, and lower NAI) and high neighborhood cancer mortality (Figure 1). The strongest association was with neighborhood guideline adherence (−0.68), which was unsurprising because, in neighborhoods that have better community‐level adherence to cancer prevention guidelines, one would expect to see lower cancer mortality. Surprisingly, the second most closely associated factor was neighborhood school trauma (−0.61), followed by neighborhood violent crime (−0.59), NAI (−0.52), and finally neighborhood health promotion (−0.49).

### 4.3 Step 3: Explaining Variation in Cancer Mortality

Due to high correlations (above 0.6) between NAI, school trauma, and crime, three multivariate regression models were tested with one of those variables along with guideline adherence and health promotion to predict cancer mortality. There were no significant spatial dependencies of the multivariate regression residuals, indicating that ordinary least squares models were appropriate (e.g., spatial regression was not required). Rather than selecting a single measure of trauma or combining them, the three trauma‐related variables were retained as separate constructs in model testing as they capture conceptually distinct dimensions of trauma: school trauma reflects child‐specific experiences within educational settings, the NAI represents broader structural and environmental conditions associated with trauma, and crime offers a simplified, single‐indicator marker of community‐level trauma. The primary model (Model 1) included guideline adherence and health promotion along with NAI. The results (Table 1) showed that should the guideline adherence score of a neighborhood increase by one full point, the cancer mortality rate in that neighborhood would go down by 16 (SE: 4.5) deaths per 100,000 people (*p* < 0.001). In this same model, an improvement by one unit on NAI was associated with a decrease in the cancer rate by 9 (SE: 3.7) deaths per 100,000 residents (*p* < 0.01). The adjusted *R* ^2^ for this model was 0.50, indicating that approximately 50% of the variance in cancer mortality can be explained by the predictors included. In two subsequent models, school trauma (Model 2) and then crime (Model 3) replaced the NAI predictor. In Model 2, school trauma played a very similar role to the model with NAI; however, the point estimate of school trauma and the resulting *R* ^2^ were both slightly larger (11.0, SE: 4.1, *p* < 0.01 and 0.52, respectively). In Model 3, crime played a very similar role to the one that NAI and school trauma had played in the previous models. The effect size of crime and the resulting *R* ^2^ were very close (10.8, SE: 4, and 0.52, respectively); however, crime reached a higher level of significance (*p* < 0.001) than previous trauma indicators, and model fit was marginally improved.

**TABLE 1:** Regression results.

	Model 1	Model 2	Model 3
Intercept	195.774*** (3.423)	196.025*** (3.378)	196.023*** (3.370)
Guideline adherence	−15.804** (4.489)	−13.640** (4.713)	−13.615** (4.690)
Health promotion	−4.400 (4.222)	−4.632 (4.166)	−5.164 (4.156)
NAI	−9.048* (3.706)		
School trauma		−10.962* (4.088)	
Violent crime			−10.810 (3.962)**
*R* ^2^	0.5378	0.5493	0.5516
Adjusted *R* ^2^	0.5048	0.5171	0.519

*Note:* Significance levels: < 0.0001= ***, 0.001 = **, 0.01 = *, 0.05 = ‘.’

Across all models, guideline adherence displayed a consistent effect on cancer mortality, confirming its significance, while health promotion was not significant in any of the three models. The unique trauma‐related predictors introduced in each model allowed for the assessment of their individual associations with cancer mortality. These results provide valuable insights into the relationships between the predictors and cancer mortality in Philadelphia neighborhoods. The adjusted *R* ^2^ values indicate that about half of the variation in cancer mortality at the neighborhood level can be explained by community‐level adult guideline adherence and area‐level trauma measures representing childhood and school trauma and exposure to violent crime.

### 4.4 Step 4: Identifying Patterns of Overlap

**FIGURE 2:**
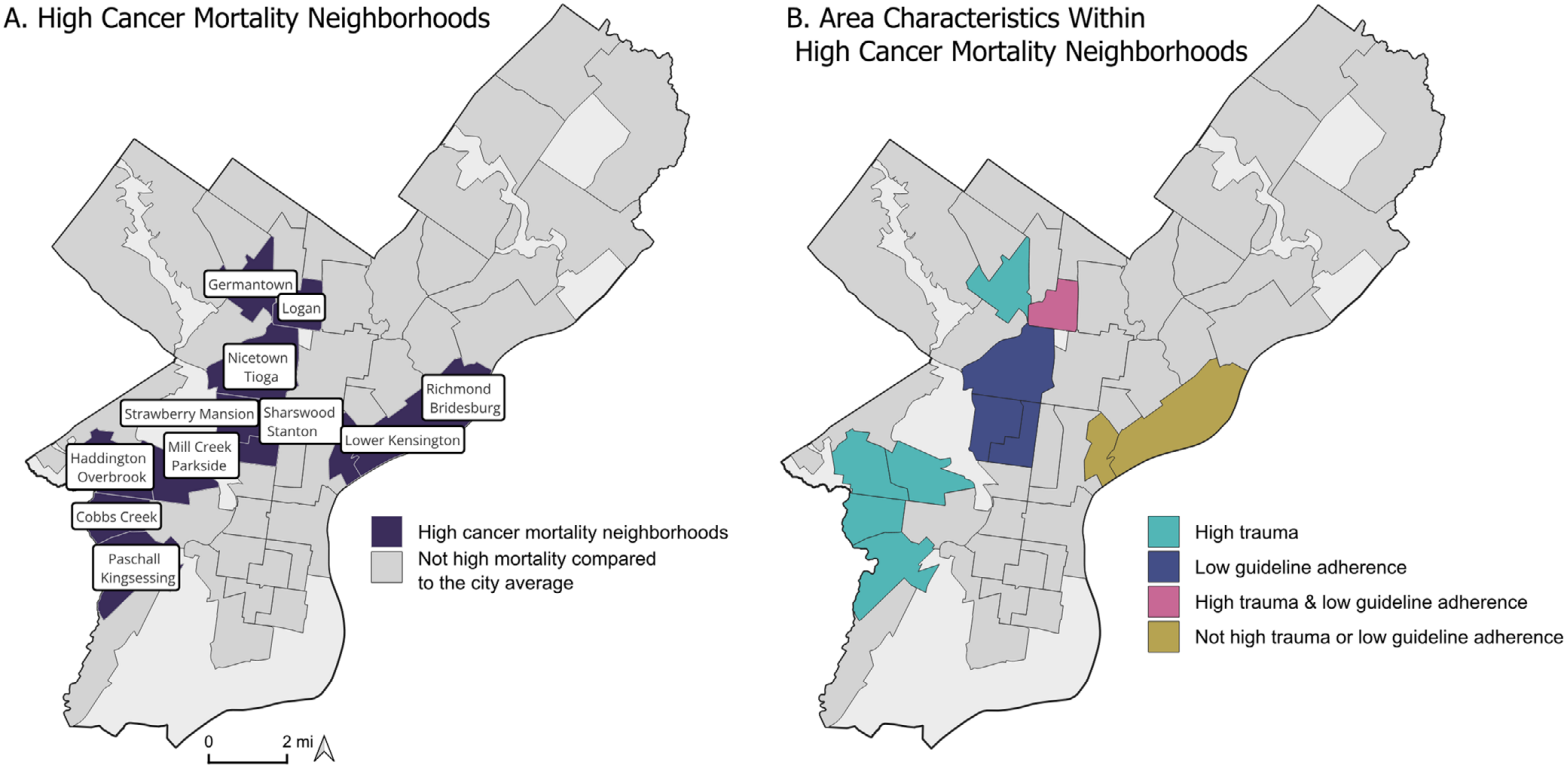
High cancer mortality neighborhoods in Philadelphia. (A) Labeled and indicated in dark blue, and (B) with prominent indicators identified. This figure displays two maps of Philadelphia neighborhoods. Most neighborhoods are displayed in gray. The same 11 neighborhoods are in color in each map. These are the neighborhoods that have higher all‐cause cancer mortality than the city overall. In the map on the left, the neighborhoods of interest are shaded in dark navy blue and each one is labeled with the neighborhood name. In the map on the right, the neighborhoods are colored to indicate the variables of interest that are high compared to the city overall. Teal indicates a high trauma measure, dark blue indicates low adherence to cancer prevention guidelines, pink indicates high trauma and low adherence to cancer prevention guidelines, and yellow indicates that the neighborhood is neither high on trauma or low on cancer adherence guidelines. (A) High cancer mortality is determined by comparing neighborhood rates to the city average. If the confidence interval of a neighborhood's cancer mortality estimate is entirely above the city average, it is considered to have a high rate of cancer mortality. (B) Only neighborhoods with high cancer mortality compared to the city overall are shown in color. For trauma and guideline adherence, more and less favorable is defined by membership in the first or fourth quartiles when considering rates of all the neighborhoods in the city. Neighborhoods with high cancer mortality appear geographically near each other. Similarly, area characteristics are frequently shared by nearby neighborhoods.

Among the 11 neighborhoods exhibiting higher cancer mortality rates than the city overall (Figure 2A), eight of them notably find themselves in the highest quartile for two to three area‐level trauma measures, which include NAI, school trauma, and crime.

Within these eight neighborhoods characterized by high cancer mortality and high trauma, only three (Nicetown–Tioga, Strawberry Mansion, Sharswood–Stanton) exhibit poor adherence to cancer prevention guidelines, while the other five (Germantown, Paschall–Kingsessing, Cobbs Creek, Haddington–Overbrook, Mill Creek–Parkside) align with the city overall in terms of adherence. As for the three neighborhoods marked by elevated cancer mortality but not considered high in trauma, one (Logan) demonstrates low adherence to cancer prevention guidelines and resides in close proximity to other neighborhoods sharing this feature of low adherence and high trauma. Conversely, the remaining two neighborhoods (Lower Kensington, Richmond–Bridesburg) are situated next to each other in a different part of the city and do not exhibit unfavorable characteristics in any of the measures employed in this analysis.

High cancer mortality neighborhoods were distinctly categorized based on their overlapping characteristics, allowing for a visual representation of these intriguing patterns (Figure 2B).

This complex interplay of factors provides valuable insights into the relationships between cancer mortality, trauma, adherence to guidelines, and crime within Philadelphia's neighborhoods.

## 5 Discussion

Among neighborhoods with the highest cancer mortality rates, four distinct profiles emerged. In one group of three neighborhoods, low adherence to cancer prevention guidelines and high trauma were observed. In another group of five neighborhoods, adherence to guidelines did not differ from the city average, but trauma levels were notably higher. A third profile is represented by one neighborhood which had low adherence to cancer prevention guidelines but did not differ from the city overall on trauma. Finally, in a fourth group of two high‐mortality neighborhoods, neither adherence to guidelines nor trauma was significantly worse than in other areas. Multivariate regression analysis indicated that neighborhood‐level adherence to cancer prevention guidelines and trauma exhibit distinct associations with cancer mortality.

These observations have two main implications. First, the findings highlight the substantial overlap between high cancer burden and high trauma exposure in Philadelphia neighborhoods. Cancer is a complex experience that can be fraught with psychological challenges and has the potential to be traumatic and/or exacerbate the effects of prior trauma, especially for individuals and families who have previously experienced non‐cancer‐related trauma [39]. Recent literature on trauma‐informed care (TIC) in oncology suggests that adopting a TIC approach has the capacity to enhance the quality of care and patient outcomes at the individual and community levels [39, 40]. However, additional research is needed to illuminate best practices for delivering trauma‐informed cancer care more broadly across a healthcare system primarily serving areas with heightened population‐level trauma.

The second implication of this work relates to how trauma is conceptualized as a public health issue. Understanding that trauma is impacted by context, this study attempted to account for some of the complexity of the manifestation of trauma by using multiple trauma‐related measures within a socio‐ecological framework. The findings of this study reveal that crime, a potentially traumatizing event, as well as school‐level measures of trauma‐related behaviors, were strongly correlated with NAI and exhibited similar explanatory abilities and spatial distributions. However, there were some differences that may impact the choice of which measure to use in future analyses, depending on a study's area of focus. For example, the school‐level trauma indicator demonstrates a stronger correlation with cancer mortality than the NAI. Furthermore, unlike violent crime and the NAI, less school trauma was positively correlated with health promotion activities. Finally, of the three related measures, violent crime explained 52% of the variation in cancer mortality when combined with adherence to cancer prevention guidelines. Despite the differences in the related measures, the high degree of similarity found between the measures related to NAI (based on ACEs measured in the built environment), school trauma (based on school‐level measures of behaviors associated with traumatized students), and violent crime (a potentially trauma‐associated event) suggests support for a broad definition of trauma in public health applications. Although the school trauma index had not been previously validated, its alignment with the other measures highlights that diverse conceptualizations of trauma are associated with similar health effects. From a public health perspective, employing a more encompassing trauma category may ensure that services reach a wider population of individuals potentially affected by trauma. This may be particularly valuable when employing a geospatial methodology to identify neighborhoods that should be prioritized for intervention.

While this analysis provides novel insights, it is not without limitations. First, the trauma measures were proxies for trauma exposure rather than direct assessments of psychological effects, which can be measured via clinical diagnoses (e.g., post‐traumatic stress disorder (PTSD)), research tools, or operationalizations (e.g., ACEs). Second, high‐quality deprivation indices such as the Area Deprivation Index were not included in this study, as the primary neighborhood‐level measure was designed to capture trauma‐related exposures that are conceptually distinct from general deprivation. Further, including another composite measure within the NAI could introduce redundancy and methodological concerns. Third, using neighborhoods as the unit of analysis ensured sufficient population size, but neighborhoods are limited in that they are spatial proxies rather than true borders, and cannot fully encapsulate residents' lived experiences of their communities. Finally, reliance on present‐day exposure measures alongside cancer mortality data limits the ability to establish temporal order between exposure and outcome. Due to the lack of data related to temporally relevant individual‐level exposures and outcomes, the analysis did not allow for an assessment of causality. The associations observed are instead intended to highlight neighborhood‐level patterns that may warrant further investigation in studies designed to examine causal pathways.

This analysis highlights overlapping geographic disparities in cancer and trauma burden, underscoring the importance of considering the local context and recognizing and nurturing community strengths [41]. In Philadelphia, community‐led organizations dedicated to the physical health and mental well‐being of youth [42], as well as the School District's trauma initiatives through partnerships, teacher training, and events, aim to promote healing and empowerment among youth impacted by trauma [43]. These efforts take place in a city where historical patterns of discriminatory housing practices and unequal access to resources have shaped socio‐ecological and health conditions [44, 45], and where neighborhoods and schools remain highly segregated [46]. As a result, neighborhood‐level analyses inevitably intersect persistent racial inequities [47], and future research is needed to explore racial disparities related to neighborhood trauma and cancer burden [48]. Building on community strengths, policymakers, healthcare systems, and academic institutions can collaborate with local community organizations where trauma and cancer burden are both high. Such partnerships may include place‐based outreach, shared educational events, and health promotion initiatives that address both trauma and cancer prevention. Sustained funding will be critical to support these efforts, and future research should evaluate their impact on screening, treatment engagement, survival, and disparity reduction.

Collectively, this research explores neighborhood‐level patterns between childhood trauma, social and physical environments, and cancer. Results expand upon prior research by characterizing these patterns at the neighborhood level, showing co‐occurrence of childhood trauma with differences in cancer prevention behaviors and cancer mortality. Findings suggest neighborhood‐level trauma measures may help identify and prioritize areas for targeted cancer prevention efforts, that integrating a trauma‐informed approach into cancer prevention and care could be valuable, and that future individual‐level studies of place‐based trauma and cancer outcomes may be warranted to address geographic cancer disparities.

## Author Contributions

**Tesla D. DuBois:** conceptualization; formal analysis; writing – original draft; methodology; visualization; data curation. **Shannon M. Lynch:** conceptualization; writing – review and editing; funding acquisition; supervision; investigation. **Jayme Banks:** writing – review and editing; data curation. **David B. Sarwer:** conceptualization; writing – review and editing. **Krista Schroeder:** writing – review and editing; data curation; conceptualization; investigation; methodology; supervision; validation.

## Ethics Statement

The authors have nothing to report.

## Consent

The authors have nothing to report.

## Conflicts of Interest

Dr. Sarwer discloses ongoing consulting relationships with NovoNordisk and Twenty30 Health. The other authors declare no conflicts of interest.

## Supporting information


**Figure S1:** Spatial distribution of measures in Philadelphia neighborhood.

## Data Availability

This study used three neighborhood‐level indices. Three of these indices were developed in prior work by members of the study team, with detailed methodology and data sources described in previously published manuscripts. The data used in those indices are publicly available; however, some datasets require purchase or are subject to data use agreements that prohibit redistribution. The fourth index was developed for this study using publicly available data from the School District of Philadelphia. Finally, crime data was downloaded from the Philadelphia Police Department. All data sources are cited in the manuscript, and links to publicly available datasets are provided where possible.
