# Supplementary material for: Associations Between Neighborhood Environment, Childhood Adversity, and Cancer Risk: A Geospatial Analysis
Source: Cancer Med. 2025 Oct 29;14(21):e71331. doi: 10.1002/cam4.71331 (PMC12572626; doi:10.1002/cam4.71331)
Supplement: Supplementary file 1 — Figure S1: Spatial distribution of measures in Philadelphia neighborhood. [file CAM4-14-e71331-s001.docx]

**Figure S1**

*Spatial Distribution of Measures in Philadelphia Neighborhood*


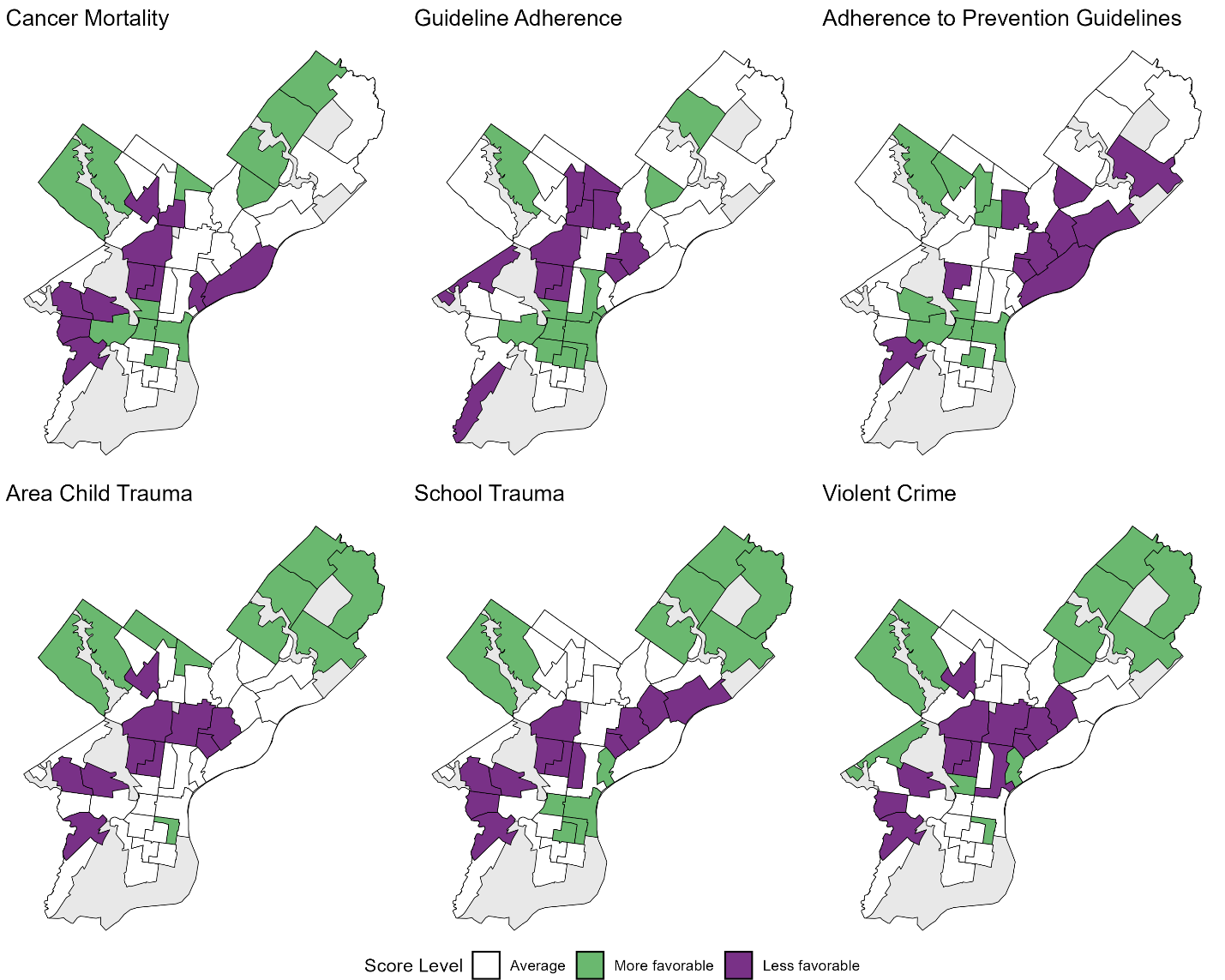


*Note:* More and less favorable are defined by membership in the first or fourth quartiles when considering rates of all the neighborhoods in the city.
